# Body weight and risk of soft-tissue sarcoma

**DOI:** 10.1038/sj.bjc.6690781

**Published:** 1999-11

**Authors:** A Tavani, M Soler, C La Vecchia, E Negri, S Gallus, S Franceschi

**Affiliations:** Istituto di Ricerche Farmacologiche ‘Mario Negri’, Via Eritrea 62, Milano, 20157, Italy; Istituto di Biometria e Statistica Medica, Università di Milano, Via Venezian 1, Milano 20133, Italy; Centro di Riferimento Oncologico, Via Pedemontana Occidentale, 33081 Aviano (PN), Italy

**Keywords:** body mass index, case–control studies, obesity, overweight, soft-tissue sarcoma

## Abstract

The relation between body mass (BMI) and soft-tissue sarcoma (STS) risk was evaluated in a case–control study from Northern Italy based on 217 incident STS and 1297 hospital controls. The risk of STS rose with BMI, with multivariate odds ratios of 3.49 (95% confidence interval (CI) 1.06–11.55) among men and 3.26 (95% CI 1.27–8.35) among women with a BMI >30 kg m^–2^ compared to those with BMI ≤ 20 kg m^–2^. © 1999 Cancer Research Campaign


*British Journal of Cancer* (1999) **81**(5), 890–892
© 1999 Cancer Research Campaign
Article no. bjoc.1999.0781

### Body weight and risk of soft-tissue sarcoma

**A Tavani[1], M Soler[1], C La Vecchia[1,2], E Negri[1], S Gallus[1] and S Franceschi[3]**

[1]Istituto di Ricerche Farmacologiche 'Mario Negri', Via Eritrea 62, 20157 Milano, Italy; [2]Istituto di Biometria e Statistica Medica, Università di Milano, Via Venezian 1, 20133 Milano, Italy; [3]Centro di Riferimento Oncologico, Via Pedemontana Occidentale, 33081 Aviano (PN), Italy

**Summary** The relation between body mass (BMI) and soft-tissue sarcoma (STS) risk was evaluated in a case–control study from Northern Italy based on 217 incident STS and 1297 hospital controls. The risk of STS rose with BMI, with multivariate odds ratios of 3.49 (95% confidence interval (CI) 1.06–11.55) among men and 3.26 (95% CI 1.27–8.35) among women with a BMI >30 kg m$^{-2}$ compared to those with BMI ≤ 20 kg m$^{-2}$. © 1999 Cancer Research Campaign

**Keywords:** body mass index; case–control studies; obesity; overweight; soft-tissue sarcoma

A population-based case–control study (Zahm et al, 1989) conducted in Kansas, USA and based on 133 cases showed that the risk of soft-tissue sarcoma (STS) increased with adult body weight, the odds ratio (OR) being 2.0 for people weighing more than 200 pounds. To provide further information on this issue, we analysed data from a large case–control study conducted in Italy.

### SUBJECTS AND METHODS

Data were derived from a case–control study of STS, conducted between 1983 and 1998 in the greater Milan area and the province of Pordenone, northern Italy (Serraino et al, 1991; Tavani et al, 1997).

Cases were 217 patients (113 men, median age 52 years, range 21–79; and 104 women, median age 54 years, range 16–79) with histologically confirmed incident (i.e. diagnosed within the year preceding the interview) STS (International Classification of Diseases IX Edition ICD-171) admitted to the cancer institutes and major teaching and general hospitals of the areas under surveillance.

Controls were 1297 patients (792 men, median age 55 years, range 17–79; and 505 women, median age 57 years, range 17–79) admitted to the same network of hospitals for acute, non-neoplastic and non-immune-related conditions. Of the comparison group, 15% were admitted for traumas, 27% for other orthopaedic disorders (such as low back pain and disc diseases), 29% for acute surgical conditions (such as acute appendicitis or strangulated hernia), and 28% for other miscellaneous illnesses (such as ear, nose and throat, eye, dental or skin disorders). On average, fewer than 4% of the eligible cases and controls refused the interview.

Trained interviewers used a structured questionnaire to collect data on socio-demographic characteristics, anthropometric measures (self-reported weight and height), personal and family history of selected medical conditions, a few selected occupational and environmental exposures, smoking status and consumption of alcohol, coffee and selected dietary items.

### Data analysis

Body mass index (BMI) was computed according to Quetelet's index (weight height$^{-2}$, kg m$^{-2}$), which is essentially an indicator of weight unrelated to height (Benn, 1971). Odds ratios (OR) with their corresponding 95% confidence intervals (CI) were derived using unconditional multiple logistic regression analysis, fitted by the method of maximum likelihood (Breslow and Day, 1980). The regression models included terms for study centre, year of recruitment, age (8 levels plus a continuous term) and education.

### RESULTS

Table 1 gives the distribution of STS cases and controls by sex according to age and BMI, and the OR of STS according to BMI. The risk of STS rose with BMI, and the OR was 3.28 in subjects with a BMI > 30 compared to those with BMI ≤ 20. The OR was 3.49 for men and 3.26 for women. The risk estimates did not change after further adjustment for employment in agriculture and radiation therapy: the OR became 3.41 and 3.53 respectively for men and women with BMI > 30 compared to those with BMI ≤ 20.

A separate analysis for broad categories of anatomical sites of STS showed no differences in the strength of association by site: the OR for BMI > 30 compared to ≤ 20 for men and women combined was 3.02 (95% CI 1.21–7.52) for STS in the upper or lower limbs (ICD 171.2 and 171.3), 3.75 (95% CI 0.94–14.96) for STS in the abdomen or pelvis (ICD 171.5 and 171.6) and 2.88 (95% CI 0.66–12.58) for those arising in other or unspecified sites.

Height was not associated with risk of STS; the OR for the highest quartile compared to the lowest one was 1.14 (95% CI 0.59–2.22) in men and 0.50 (95% CI 0.25–0.99) in women.

### DISCUSSION

The results of this study suggest a direct association between risk of STS and BMI in men and women and no relation with height, in agreement with the findings of Zahm et al (1989).

*Received 5 March 1999*
*Revised 19 April 1999*
*Accepted 5 May 1999*

*Correspondence to:* A Tavani

**Table 1** Distribution of 217 cases of soft-tissue sarcoma (STS) and 1297 controls, and corresponding odds ratios with 95% confidence intervals, according to age and body mass index (BMI); Italy, 1983–1998

| | STS | | Controls | | Odds ratios (95% confidence intervals)[a] | | |
|---|---|---|---|---|---|---|---|
| | Men | Women | Men | Women | Men | Women | All |
| Age (years) | | | | | | | |
| <40 | 26 | 13 | 156 | 106 | | | |
| 40–49 | 23 | 21 | 134 | 65 | | | |
| 50–59 | 32 | 33 | 187 | 104 | | | |
| 60–69 | 18 | 25 | 228 | 139 | | | |
| ≥70 | 14 | 12 | 87 | 91 | | | |
| BMI ($kg/m^2$)[b] | | | | | | | |
| ≤20 | 4 | 10 | 71 | 96 | 1[c] | 1[c] | 1[c] |
| >20–25 | 56 | 36 | 292 | 199 | 3.38 (1.15–9.90) | 1.66 (0.73–3.76) | 2.38 (1.28–4.44) |
| >25–30 | 39 | 38 | 355 | 151 | 1.63 (0.54–4.88) | 2.47 (1.07–5.69) | 1.93 (1.02–3.67) |
| >30 | 14 | 19 | 73 | 59 | 3.49 (1.06–11.55) | 3.26 (1.27–8.35) | 3.28 (1.61–6.69) |
| $\chi^2$, trend | | | | | 0.02 (p=0.900) | 7.69 (p=0.006) | 4.78 (p=0.029) |

[a]Estimates from multiple logistic regression equations, including terms for study centre, year of recruitment, age and education. The ‘All’ category was further allowed for sex. [b]The sum does not add up to the total because of some missing values. [c]Reference category.

Overweight and obesity are major public health issues and are associated with several chronic diseases and shorter life expectancy (Manson et al, 1987), probably through multiple biological mechanisms. Obese subjects were at elevated risk of leukaemias and lymphomas in the American Cancer Society cohort study conducted between 1959 and 1972 (Lew and Garfinkel, 1979). It is biologically plausible that overweight and obese people may be at higher risk of STS as, besides radiation, selected chemicals and some drugs and infectious agents (Zahm et al, 1996; Zahm and Fraumeni, 1997), herbicides and pesticides, particularly those containing 2,3,7,8-tetrachlorodibenzo-p-dioxin, chlorophenols and dibenzofurans, have been associated to STS risk (Hoar et al, 1986; Kang et al, 1987; Woods et al, 1987; Reif et al, 1989; Saracci et al, 1991; Bertazzi et al, 1993; Kogevinas et al, 1997; Hoppin et al, 1998), and most of these chemicals accumulate in human adipose tissue (Patterson et al, 1996; Kang et al, 1991; Orban et al, 1994). Very lean subjects may be selectively at low risk because of inherent characteristics of their adipose tissue, and of its specific role in STS pathogenesis. Moreover, immunological and hormonal imbalances in obese subjects have been reported, including changes in leptin (Friedman, 1997) and insulin growth factors, that regulate metabolism and play an important role in proliferation and apoptosis of several cell types (Daughaday, 1990; Tritos and Mantzoros, 1998), and that have been related to the risk of several neoplasms (Mantzoros et al, 1997; Petridou et al, 1999).

A possible source of bias in this study is the misclassification of body mass (Millar, 1986; Nieto-Garcia et al, 1990), as self-reported measures tend to overestimate height and underestimate weight (Palta et al, 1982; Stewart, 1982; Millar, 1986; Nieto-Garcia et al, 1990). However, such information bias is likely to be similar for cases and controls, and cannot explain the strong association observed. Hospital controls may differ from the general population in several respects (Breslow and Day, 1980). However, overweight and obesity are related to an increased prevalence of several chronic diseases (Negri et al, 1988; Pagano et al, 1997), so the use of hospital controls should, if anything, underestimate the true association (Breslow and Day, 1980).

A major difficulty in this and other epidemiological studies on STS is the relatively low incidence and wide heterogeneity of this neoplasm, as aetiological differences by subsite and cell type have been suggested (Zahm et al, 1996; Zahm and Fraumeni, 1997).

The prevalence of overweight and obesity has been rising over the last few years in Italy (Pagano et al, 1997) and other Western countries (Galuska et al, 1996). This might partly explain the increased trends in incidence and mortality from STS (La Vecchia et al, 1992; Ross et al, 1993).

Thus, these data suggest that BMI is directly related to STS risk, and offer another reason for public health interventions to reduce overweight and obesity.

### ACKNOWLEDGEMENTS

This work was conducted with the contribution of the Italian Association for Cancer Research, Milan. Dr Maria Soler was recipient of a ‘Zambon’ fellowship awarded by the Zambon Group, Spain. The authors thank Mrs J Baggott and MP Bonifacino for editorial assistance.

### REFERENCES

Benn RT (1971) Some mathematical properties of weight-for-height indices used as measures of adiposity. *Br J Prev Soc Med* **25**: 42–50

Bertazzi PA, Pesatori AC, Consonni D, Tironi A, Landi MT and Zocchetti C (1993) Cancer incidence in a population accidentally exposed to 2,3,7,8-tetrachlorodibenzo-para-dioxin. *Epidemiology* **4**: 398–406

Breslow NE and Day NE (1980) *Statistical Methods in Cancer Research. Vol. 1. The Analysis of Case–control Studies*. IARC Scientific Publication **32**: IARC: Geneva, pp. 1–279

Daughaday WH (1990) The possible autocrine/paracrine and endocrine roles of insulin-like growth factors of human tumors. *Endocrinology* **127**: 1–4

Friedman JM (1997) The alphabet of weight control. *Nature* **385**: 119–120

Galuska DA, Serdula M, Pamuk E, Siegel PZ and Byers T (1996) Trends in overweight among US adults from 1987 to 1993: a multistate telephone survey. *Am J Public Health* **86**: 1729–1735

Hoar SK, Blair A, Holmes FF, Boysen CD, Robel RJ, Hoover R and Fraumeni JF Jr (1986) Agricultural herbicide use and risk of lymphoma and soft-tissue sarcoma. *JAMA* **256**: 1141–1147

Hoppin JA, Tolbert PE, Herrick RF, et al (1998) Occupational chlorophenol exposure and soft tissue sarcoma risk among men aged 30–60 years. *Am J Epidemiol* **148**: 693–703

Kang H, Enziger F, Breslin P, Feil M, Lee Y, Shepard B (1987) Soft tissue sarcoma and military service in Vietnam: a case–control study. *J Natl Cancer Inst* **79**: 693–699

Kang HK, Watanabe KK, Breen J, et al. (1991) Dioxins and dibenzofurans in adipose tissue of US Vietnam veterans and controls. *Am J Public Health* **81**: 344–349

Kogevinas M, Becher H, Benn T, et al (1997) Cancer mortality in workers exposed to phenoxy herbicides, chlorophenols, and dioxins. An expanded and updated international cohort study. *Am J Epidemiol* **145**: 1061–1075

La Vecchia C, Lucchini F, Negri E, Boyle P, Maisonneuve P and Levi F (1992) Trends of cancer mortality in Europe, 1955–1989: II, respiratory tract, bone, connective and soft tissue sarcomas, and skin. *Eur J Cancer* **28**: 514–599

Lew EA and Garfinkel L (1979) Variations in mortality by weight among 750,000 men and women. *J Chron Dis* **32**: 563–576

Manson JE, Stampfer MJ, Hennekens CH and Willett WC (1987) Body weight and longevity. A reassessment. *JAMA* **257**: 353–358

Mantzoros CS, Tzonou A, Signorello LB, et al (1997) Insulin-like growth factors 1 in relation to prostate cancer and benign prostatic hyperplasia. *Br J Cancer* **76**: 1115–1118

Millar WJ (1986) Distribution of body weight and height: comparison of estimates based on self-reported and observed measures. *J Epidemiol Community Health* **40**: 319–323

Negri E, Pagano R, Decarli A and La Vecchia C (1988) Body weight and the prevalence of chronic diseases. *J Epidemiol Community Health* **42**: 24–29

Nieto-Garcia FJ, Bush TL and Keyl PM (1990) Body mass definitions of obesity: sensitivity and specificity using self-reported weight and height. *Epidemiology* **1**: 146–152

Orban JE, Stanley JS, Schwemberger JG and Remmers JC (1994) Dioxins and dibenzofurans in adipose tissue of the general US population and selected subpopulations. *Am J Public Health* **84**: 439–445

Pagano R, La Vecchia C, Decarli A, Negri E and Franceschi S (1997) Trends in overweight and obesity among Italian adults, 1983 through 1994. *Am J Public Health* **87**: 1869–1870

Palta M, Prineas RJ, Berman R and Hannan P (1982) Comparison of self-reported and measured height and weight. *Am J Epidemiol* **115**: 223–230

Patterson DG Jr, Hoffman RE, Needham LL, et al. (1986) 2,3,7,8-tetrachlorodibenzo-*p*-dioxin levels in adipose tissue of exposed and control persons in Missouri. An interim report. *JAMA* **256**: 2683–2686

Petridou E, Dessypris N, Spanos E, et al. (1999) Insulin-like growth factor-I and binding protein-3 in relation to childhood leukaemia. *Int J Cancer* **80**: 494–496

Reif J, Pearce N, Kawachi I and Fraser J (1989) Soft-tissue sarcoma, non-Hodgkin's lymphoma and other cancers in New Zealand forestry workers. *Int J Cancer* **43**: 49–54

Ross JA, Severson RK, Davis S and Brooks JJ (1993) Trends in the incidence of soft tissue sarcomas in the United States from 1973 through 1987. *Cancer* **72**: 486–490

Saracci R, Kogevinas M, Bertazzi PA, et al. (1991) Cancer mortality in workers exposed to chlorophenoxy herbicides and chlorophenols. *Lancet* **338**: 1027–1032

Serraino D, Franceschi S, Talamini R, Frustaci S and La Vecchia C (1991) Non-occupational risk factors for adult soft-tissue sarcoma in northern Italy. *Cancer Causes Control* **1**: 157–164

Stewart AL (1982) The reliability and validity of self-reported weight and height. *J Chron Dis* **35**: 295–309

Tavani A, Pregnolato A, Negri E, et al. (1997) Diet and risk of lymphoid neoplasms and soft tissue sarcomas. *Nutr Cancer* **27**: 256–260

Tritos NA and Mantzoros CS (1998) Recombinant human growth hormone: old and novel uses. *Am J Med* **105**: 44–57

Woods JS, Polissar L, Severson RK, Heuser LS and Kulander BG (1987) Soft tissue sarcoma and non-Hodgkin's lymphoma in relation to phenoxyherbicide and chlorinated phenol exposure in Western Washington. *J Natl Cancer Inst* **78**: 899–910

Zahm SH, Blair A, Holmes FF, Boysen CD, Robel RJ and Fraumeni JF Jr (1989) A case–control study of soft-tissue sarcoma. *Am J Epidemiol* **130**: 665–674

Zahm SH and Fraumeni JF Jr (1997) The epidemiology of soft tissue sarcomas. *Semin Oncol* **24**: 504–514

Zahm SH, Tucker MA and Fraumeni JF Jr (1996) Soft tissue sarcomas. In: *Cancer by Tissue Origin*, Schottenfeld D and Fraumeni JF Jr (eds) pp. 984–999. Oxford University Press: New York

*© 1999 Cancer Research Campaign*
